# Ribitol dose-dependently enhances matriglycan expression and improves muscle function with prolonged life span in limb girdle muscular dystrophy 2I mouse model

**DOI:** 10.1371/journal.pone.0278482

**Published:** 2022-12-01

**Authors:** Bo Wu, Morgan Drains, Sapana N. Shah, Pei Juan Lu, Victoria Leroy, Jessalyn Killilee, Raegan Rawls, Jason D. Tucker, Anthony Blaeser, Qi Long Lu

**Affiliations:** McColl-Lockwood Laboratory for Muscular Dystrophy Research, Atrium Health Musculoskeletal Institute, Carolinas Medical Center, Charlotte, North Carolina, United States of America; UAB School of Medicine, UNITED STATES

## Abstract

Limb Girdle Muscular Dystrophy 2I (LGMDR9) is one of the most common LGMD characterized by defects in glycosylation of α-dystroglycan (matriglycan) resulting from mutations of Fukutin-related protein (FKRP). There is no effective therapy currently available. We recently demonstrated that ribitol supplement increases levels of matriglycan in cells *in vitro* and in FKRP-P448L (*P448L*) mutant mouse model through drinking water administration. To be clinically relevant, we have now conducted a dose-escalating efficacy study by gavage in *P448L* mutant mice. Six months of ribitol treatment daily significantly rescued functions of skeletal, respiratory, and cardiac muscles dose-dependently. This was associated with a dose dependent increase in matriglycan and improvement in muscle pathology with reductions in muscle degeneration, inflammatory infiltration and fibrosis. Importantly, ribitol significantly increased life span and muscle functions of the female animals receiving treatment from 10 months of age. The only observed side effect was gastrointestinal tract bloating with loose stool and this effect is also dose dependent. The results validate the mechanism that ribitol as a pre-substrate of glycosyltransferase is able to compensate for the decreased function of mutant FKRP with restoration of matriglycan expression and provide a guidance for future clinical trial design.

## Introduction

Mutations in Fukutin-related protein (FKRP) cause muscular dystrophy with wide range of disease phenotypes from mild limb girdle muscular dystrophy 2I (LGMDR9) to severe congenital muscular dystrophy (CMD), Walker-Warburg syndrome (WWS), and muscle-eye-brain (MEB) disease [1–3]. The common features of the diseases are progressive muscle degeneration and loss of function including both skeletal and cardiac muscles. Consequently, patients gradually lose mobility with impairment, and ultimately failure of respiratory and cardiac functions [4, 5]. The severe CMD, WWS and MEB have defects in central nerve and optical systems with developmental delay and cognitive impairment [1, 2]. Currently no treatment is available although several experimental therapies are being tested pre-clinically [6, 7].

The biochemical alteration underlining muscle weakness of the diseases is hypoglycosylation of alpha-dystroglycan (α-DG). Alpha-DG is a peripheral membrane protein glycosylated with *O*-linked glycans capable of binding to laminin as well as other extracellular matrix (ECM) proteins, including agrin, perlecan, neurexin and pikachurin [8–13]. The interaction of α-DG with ECM proteins is critical for maintaining muscle integrity. The structure of the laminin-binding *O*-mannosylated glycan (matriglycan) has recently been delineated with individual enzymes identified [14–16]. FKRP functions as a glycosyltransferase using CDP-ribitol as its substrate to add ribitol-5-phosphate (R-5-P) to the backbone of the *O*-mannosylated glycan. This permits further addition of laminin-binding GlcA-Xyl dimer repeats. Recent studies also identified isoprenoid synthase domain containing (ISPD) as a cytidyltransferase producing CDP-ribitol [16, 17]. Gerin et al. further demonstrated that ribitol treatment of ISPD-deficient cells leads to an increase of CDP-ribitol levels and partially corrects the defect in matriglycan synthesis [16]. The authors also reported that supply of ribitol can increase matriglycan in wild-type cells. We reported earlier that FKRP mutations such as *P448L* associated with severe CMD phenotype retain partial biological function and are capable of maintaining up to near normal levels of matriglycan in new-born and regenerating fibers [18, 19]. We recently reported that ribitol is able to partially restore matriglycan in the dystroglycanopathy model with mutations in *FKRP* gene [20–22]. Administration of ribitol within drinking water increases levels of R-5-P and CDP-ribitol and restores therapeutic levels of matriglycan in skeletal and cardiac muscles.

However, administration of ribitol by drinking water *ad libitum* as a treatment for FKRP-related muscular dystrophies is not clinically applicable. Furthermore, effective dose range of ribitol and treatment regime need to be established for clinical trials. To answer these questions, we have conducted a dose range study with ribitol delivered through daily gavage to the FKRP-P448L mutant mice. Efficacy of treatment was examined in two age groups, representing the early and later stages in disease progression. Ribitol treatment enhances levels of matriglycan significantly in both cardiac and skeletal muscles with improved pathology and muscle functions. Treatment given at a later stage of the disease is able to prolong the life span. These results, together with the fact that ribitol is a natural metabolite without significant side effect at high doses, warrants further development by clinical trials as a novel therapy to FKRP-related muscular dystrophy.

## Materials and method

### Animal care

All animal studies were approved by the Institutional Animal Care and Use Committee (IACUC) of Carolinas Medical Center, Atrium Health. All mice were housed in the vivarium of Carolinas Medical Center following animal care guidelines of the institute. Animals were ear tagged prior to group assignment. Food and water were available *ad libitum* during all phase of the study. Body weight was measured daily for accurate gavage and minoring mouse condition.

### Mouse model and experimental procedure

*FKRP-P448L* (*P448L*) mutant mice (*C57BL/6N* background) containing a homozygous missense mutation (*c*.*1343C>T*, p.Pro448Leu) were generated by the McColl-Lockwood Laboratory for Muscular Dystrophy Research [21, 22]. *C57BL/6* (wild-type/C57) mice were purchased from Jackson Laboratory. Ribitol was purchased from Sigma (A5502 Adonitol, ≥98%, Sigma, St. Louis) and dissolved in saline to desired concentrations as 100ul/10g mouse body weight. Three week and 10 month old *P448L* mice were treated with ribitol by gavage with the following doses and regimes: 0.5g/kg, 2g/kg, 5g/kg and 10g/kg body weight once a day. Additionally, 10g/kg daily dose was also administrated as 5g/kg twice a day and 3.3g/kg three times a day for comparison with single dose treatment. Twenty mice (with more than 8 mice for each sex) were randomly assigned to either treatment or control groups. Age-matched *P448L* and wild-type *C57BL/6* mice were gavaged with the same volume of saline as controls. Regular muscle function tests were performed, and the animals were euthanized by cervical spine dislocation under isoflurane anesthesia after 6 months (for the groups starting 3 weeks of age) or longer (for the groups starting 10 months of age) of treatment and tissues including heart, diaphragm, TA, quadriceps, liver, spleen and kidney were collected for analyses (S1 Fig in S1 File).

### Immunohistochemical and western blot analysis

Tissues were dissected and snap-frozen in dry-ice-chilled-2-methylbutane. For immunohistochemical detection of matriglycan, muscle cross sections of 6 μm of thickness were prepared from treated and saline control mice. Slides were first fixed in ice cold Ethanol:Acetic acid (1:1) for 1 min, blocked with 10% normal goat serum (NGS) in 1xTris-buffer saline (TBS) for 30 min at room temperature, and incubated overnight at 4°C with primary mouse monoclonal antibody IIH6C4 (EMD Millipore) (1:500) against matriglycan on α-DG. Negative controls received 10% normal goat serum in 1xTBS without primary antibody. Sections were washed and incubated with secondary AlexaFluor 488 or 594 goat anti-mouse IgM (Invitrogen) (1:500) at room temperature for 2 hr. Sections were then washed and finally mounted with fluorescence mounting medium (Dako) containing 1x DAPI (4’,6’-diamidino -2-phenylindole) for nuclear staining. Immunofluorescence was visualized using an Olympus BX51/BX52 fluorescence microscope (Opelco) and images were captured using the Olympus DP70 digital camera system (Opelco). Slides were examined in a blind manner by the investigators.

For western blot analysis, sections cut from tissues were homogenized in extraction buffer (50 mM Tris-HCl pH 8.0, 150 mM NaCl, and 1% Triton X-100), supplemented with 1x protease inhibitor cocktail (Sigma-Aldrich). Protein concentration was quantified by Bradford assay (Bio-Rad DC protein assay). Eighty μg of protein was loaded on an 4–15% Bio-Rad Mini-PROTEAN TGX gel (Bio-Rad) and immunoblotted. Total protein loaded from *C57* mice was half of the amount loaded for the *P448L* mice. Nitrocellulose membranes (Bio-Rad) were blocked with 5% milk in 1xPBS for 2 hr at room temperature and then incubated with the following primary antibodies overnight at 4°C: IIH6C4 (1:2000), and α-actin (Sigma) (1:1000). Horseradish peroxidase (HRP)-conjugated secondary antibodies were incubated for 2 hr at room temperature. All blots were developed by electrochemiluminescence immunodetection (PerkinElmer). ImageJ software was used for IIH6C4 band quantification from western blot.

### Histopathological and morphometric analysis

Sections of 6 μm of thickness were processed for hematoxylin and eosin (H&E) and Masson’s Trichrome staining following standard procedures. Muscle cross-sectional fiber equivalent diameter were determined from tibialis anterior (TA) and quadriceps stained with H&E using MetaMorph v7.7 Software (Molecular Devices). Percentage of centrally nucleated myofibers were manually quantified from the same tissue sections stained with H&E. Fibrotic area represented by blue staining in the Masson’s Trichrome stained sections was quantified from heart, diaphragm, TA and quadriceps using ImageJ software. For all the morphometric analyses, a total of 500 fibers from five representative 20X magnification images per each muscle per animal were used.

### Metabolite extraction from muscle tissues and LC/MS-MS analysis

Ribitol was purchased from Sigma (A5502). R-5-P and CDP-ribitol were synthesized by Z Biotech (Aurora, CO). Muscle tissues were collected, and blinded samples were subjected to the following procedure. Thirty to 80 μg of frozen tissue samples was homogenized with 400 μl of MeOH:Acetonitrile (ACN) (1:1) and then centrifugated for 5 min at 10,000 rpm. The supernatants were removed, transferred to individual wells of 96-well plate, and analyzed by LC/MS-MS. An Applied Biosystems Sciex 4000 (Applied Biosystems; Foster City, CA) equipped with a Shimadzu HPLC (Shimadzu Scientific Instruments, Inc.: Columbia, MD) and Leap auto-sampler (LEAP Technologies; Carrboro, NC) were used to detect ribitol, R-5-P and CDP-ribitol from tissue samples and synthetic compounds. The metabolites were separated on a silica gel column (Hypersil Silica 250 x 4.6 mm, 5 micron particle size) using solvent A: water, 10mM NH_4_OAc, 0.1% formic acid and solvent B: MeOH:ACN (1:1). The following gradient was used: 0–12 min, 5% buffer B; 13–14 min, 95% buffer B, 15–17 min, 5% buffer B. Under these conditions, ribitol, R-5-P and CDP-ribitol eluted at 8.3 min, 7.5 min and 8.9 min, respectively. The metabolites were analyzed using electrospray ionization mass spectrometry operated in positive ion mode, ESI +. Compounds concentration in tissue samples were determined based on standard curves prepared by serial dilutions (200–0.01 μM) of each of the compound in MeOH:ACN (1:1).

### Grip strength and treadmill test

Grip strength was assessed using a grip strength meter consisting of horizontal forelimb mesh and an angled hind limb mesh (Columbus Instruments, Columbus, OH). Five successful forelimb and hind limb strength measurements within 2 minutes were recorded, and data were normalized to body weight (BW). Treadmill test was performed on LE8700 treadmill (Panlab/Harvard Apparatus, Barcelona, Spain) supplied with shock grids mounted at the back of the treadmill, which delivered a 0.2 mA current to provide motivation for exercise. The end points were mice exhaustion, as demonstrated by the animal remaining on the shock grid for 10 consecutive seconds without getting off or 50% on/off within 1-minute period (for details, see reference 6).

### Whole body plethysmography

Respiratory functional analysis in conscious, freely moving mice were measured using a whole-body plethysmography technique. The plethysmograph apparatus (Emka Technologies, Falls Church, VA) was connected to a ventilation pump for maintaining a constant air flow, a differential pressure transducer, a usbAMP signal amplifier, and a computer running EMKA iox2 software with the respiratory flow analyzer module, which was used to detect pressure changes due to breathing and recording the transducer signal. After calibration, mice were placed inside the “free moving” plethysmograph chamber and allowed to acclimate for 5 min to minimize any effects of stress related changes in ventilation. Resting ventilation was measured for a duration of 15 min after the acclimation period. Body temperatures of mice were assumed to be 37°C and to remain constant during the ventilation protocol.

### Echocardiogram

Echocardiogram was performed using the BioscanSonixTablet Ultrasound System (Analogic Ultrasound, Peabody, MA). Detailed method was published by Blaeser et al. [23].

### Statistical analysis

All the results were expressed as means + SEM. One-Way ANOVA was used for comparing treatments with control group. Differences were considered statistically significant at *p* ≤ 0.05 (*).

## Results

### Assessment for maximum tolerable daily dose of ribitol in *P448L* mice

Our earlier study of ribitol treatment by drinking water in *P448L* mutant mice suggests that up to 10% ribitol ad libitum was safe [20]. By measuring the amount of water consumed daily (about 1 ml water consumed by a mouse of 10g body weight), 10% ribitol in drinking water delivers roughly 10g ribitol per kg body weight daily. We therefore first examined the effect of ribitol by gavage with 10g/kg once a day for 1 month in *P448L* mutant mice aged 4–6 weeks old. All 10 mice survived with the treatment although a majority displayed clearly visible signs of diarrhea and gastrointestinal (GI) bloating, which was confirmed at autopsy (S2A Fig in S1 File). No significant difference was observed in body weight, or in serum markers for liver and kidney function between ribitol treated and saline-treated control mice (S2B Fig in S1 File). Immunohistochemistry showed an enhanced expression of matriglycan in all three types of muscles, heart, diaphragm, and TA (S2C Fig in S1 File). We also measured the blood glucose levels within 48 hours after single dose gavage of 10g ribitol/kg body weight. No significant difference was observed. (S2D Fig in S1 File). We ultimately decided to use the maximum daily 10g/kg dose, as further increase in dosage to 20g/kg resulted in severe GI effects and morbidity.

### Early treatment of ribitol dose-dependently improved matriglycan levels in muscles of *P448L* mutant mice

**Fig 1:**
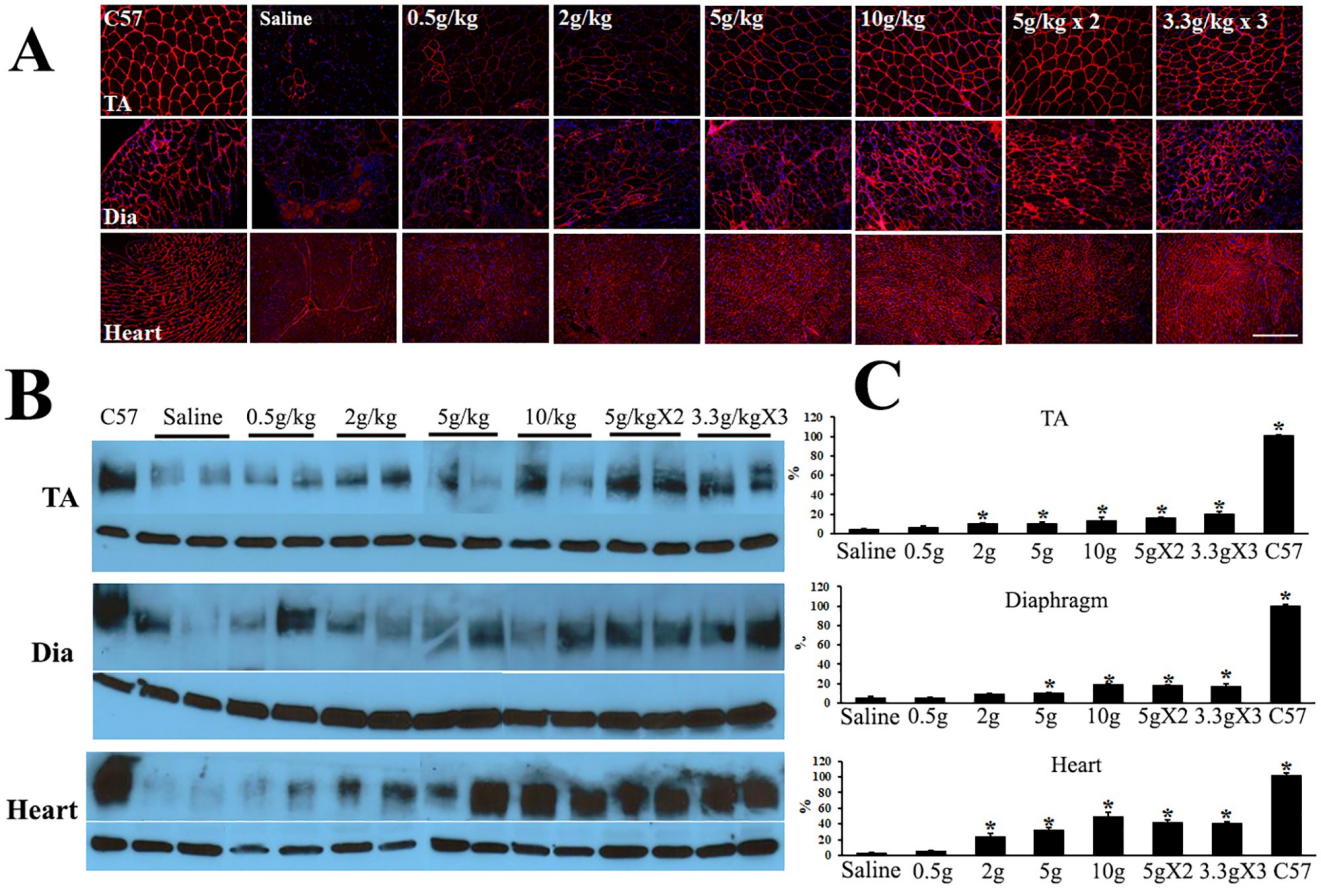
Six-month ribitol treatment enhances matriglycan expression dose-dependently in *P448L* FKRP mutant mice. (A) IIH6 immunohistochemistry for the detection of matriglycan. Saline treated as control. TA, Tibialis anterior. Dia, diaphragm. (B) Western blots for the detection of matriglycan with IIH6 antibody (with broad bands). Two samples for each dose are shown. The lower panels are α-actin as loading control. (C) measurement of the matriglycan bands by NIH ImageJ with C57 as 100% control. Saline, saline treated; 0.5g, 0.5g/kg; 5gX2, 5k/kg twice a day; 3gX3, 3g/Kg 3 times a day. * P<0.*05 n* = 3 compared to the saline control group. Scale bar = 150 μM.

To determine the effective dose range, we examined 4 daily doses of ribitol by gavage, from 0.5g/kg, 2g/kg, 5kg/kg to 10g/kg body weight starting at 3 weeks of age in the *P448L* mutant mice. The lower 3 doses were delivered once a day, whereas the 10g/kg was delivered in 3 different regimes, once daily, 5g/kg twice a day and 3.3g/kg three times a day for comparison. Six-month treatment dose-dependently and significantly enhanced the levels of matriglycan expression as illustrated in Fig 1. Treatment with 0.5g/kg did not produce clearly identifiable enhancement in matriglycan levels examined by immunohistochemistry with IIH6 antibody although weak membrane signal was present. However, clearly definable membrane staining for matriglycan was detected with the 2g/kg ribitol treatment in all three types of muscles (Fig 1A & 1B). Noticeably, the weak membrane signal in the majority of muscle fibers was rather homogenous in intensity within all examined muscles. Signal intensity for matriglycan was further enhanced with 5g/kg ribitol and reached the highest levels with 10g/kg ribitol, but the difference between the 2 highest dose groups was limited. No clear difference was observed between the 3 different regimes of 10g/kg ribitol. Interestingly, more than 90% of muscle fibers in all treated groups were stained weakly to clearly positive from lower dose to higher dose respectively (Fig 1A). The dose dependent, but not regime-related matriglycan enhancement was confirmed by western blot measurement (Fig 1B and 1C). Also noticeable was consistent high levels of matriglycan in the cardiac muscle with 5g and 10g/kg treatments (Fig 1C).

### Ribitol treatment for 6 months improves muscle pathology and reduced serum MCK levels

**Fig 2:**
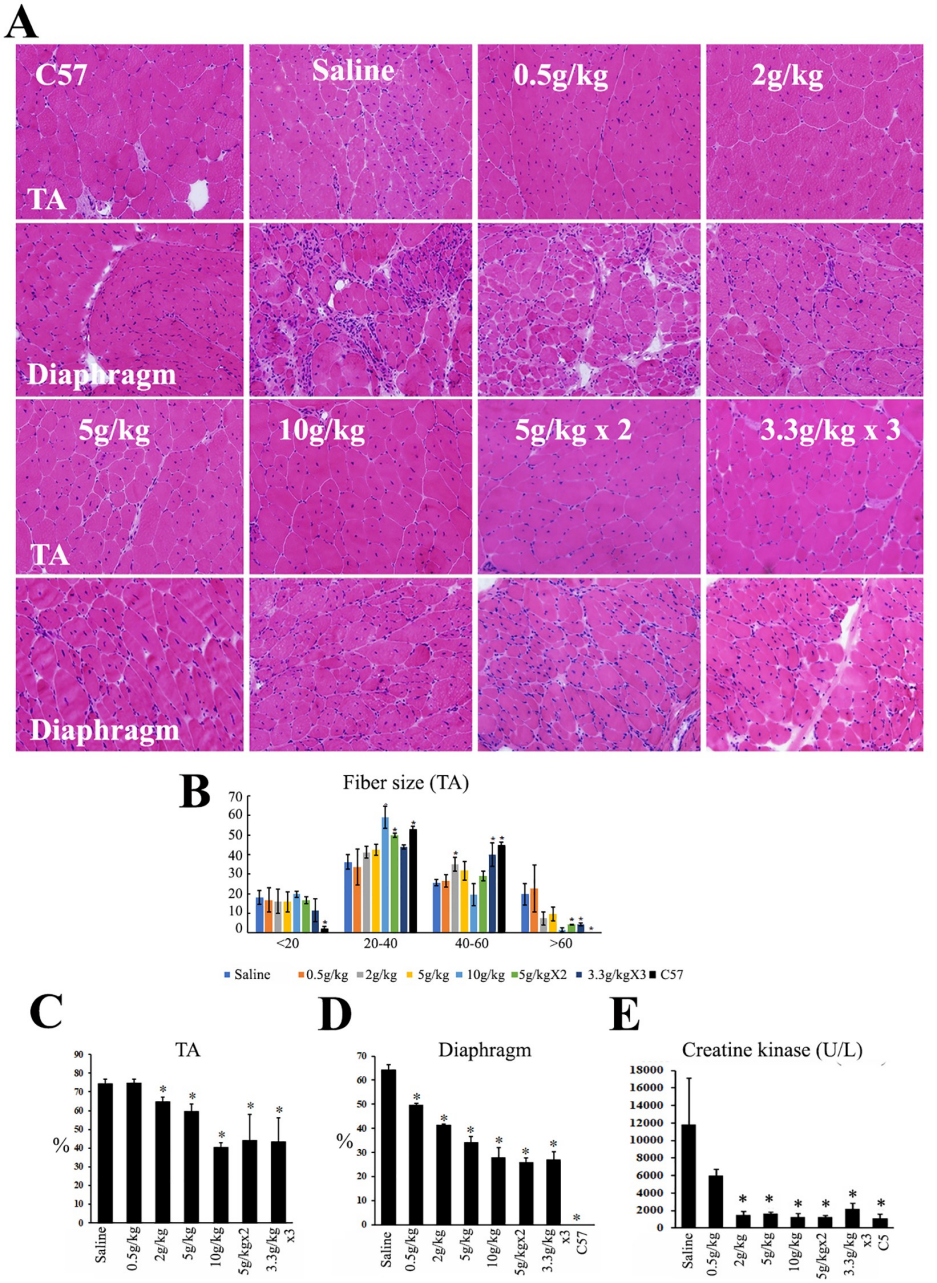
Dose-dependent improvement in muscle pathology of *P448L* mutant mice after ribitol treatment. (A) H&E staining of skeletal muscles, Tibialis anterior (TA) and diaphragm. (B) Fiber size (μM in diameter) distribution as a percent of the total fibers. *n* = 5. (C) and (D) Percentage of centrally nucleated fibers in TA and diaphragm. *n* = 5. (E) Serum levels of creatine kinase. *n* = 10. * *P<0*.*05* when compared with saline-treated control.

Enhanced expression of matriglycan after ribitol treatment was associated with a dose-dependent improvement in muscle pathology. As illustrated in Fig 2, untreated *P448L* mutant TA and diaphragm muscles showed large foci of fiber degeneration, heavy inflammatory infiltration, and high percentage of centrally nucleated fibers (CNF) with large variation in fiber size. Treatment with 0.5g/kg reduced the area with heavy infiltration although foci of small-sized regenerating fibers remained clearly observed (Fig 2A). Increasing dose of ribitol from 2g/kg to 10g/kg reduced the area of small regenerating fibers and infiltration. This was supported by a more homogeneous fiber size distribution, with less percentage of large fibers as described in Fig 2B. Furthermore, percentage of CNF in the treated skeletal muscles decreased dose-dependently (Fig 2C and 2D). Histological improvement was supported by the similarly dose-dependent decrease in serum creatine kinase levels (Fig 2E). Histological change was not clearly detected in cardiac muscle with ribitol treatment (S3 Fig in S1 File).

**Fig 3:**
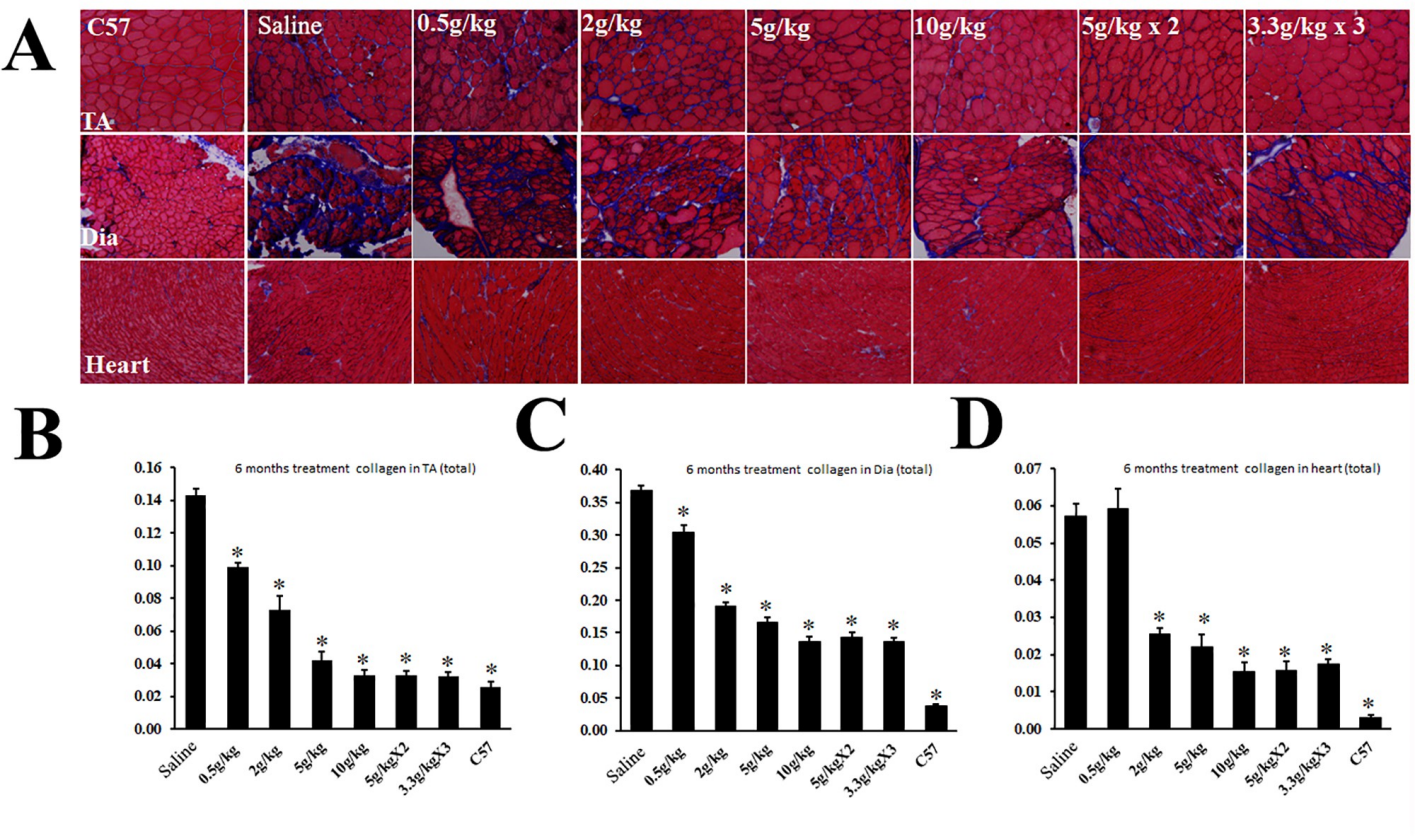
Masson Trichrome staining for the measurement of fibrotic areas in the 6-month ribitol-treated *P448L* mutant mice. (A) Microscopic image of the staining with blue color representing fibrotic areas and red color the remaining muscle fibers. (B-D) Measurement of fibrotic areas with NIH ImageJ. Y axis represents percentage of fibrotic area within the total section area (1 equals to 100% of area measured). * *P<0*.*05 n* = 5 when compared with saline-treated control.

Improvement in muscle pathology was most visibly demonstrated by Masson Trichrome staining for the measurement of fibrotic areas within the diseased muscles. A dose-dependent and statistically significant decrease in fibrotic areas was detected in all three muscles, TA, diaphragm, and heart when compared to the corresponding saline-treated control muscles (Fig 3). However, no difference was detected within 3 delivery regimes of the same 10g/kg daily dose.

### Effects of six-month ribitol treatments on functions of skeletal and cardiac muscles

**Fig 4:**
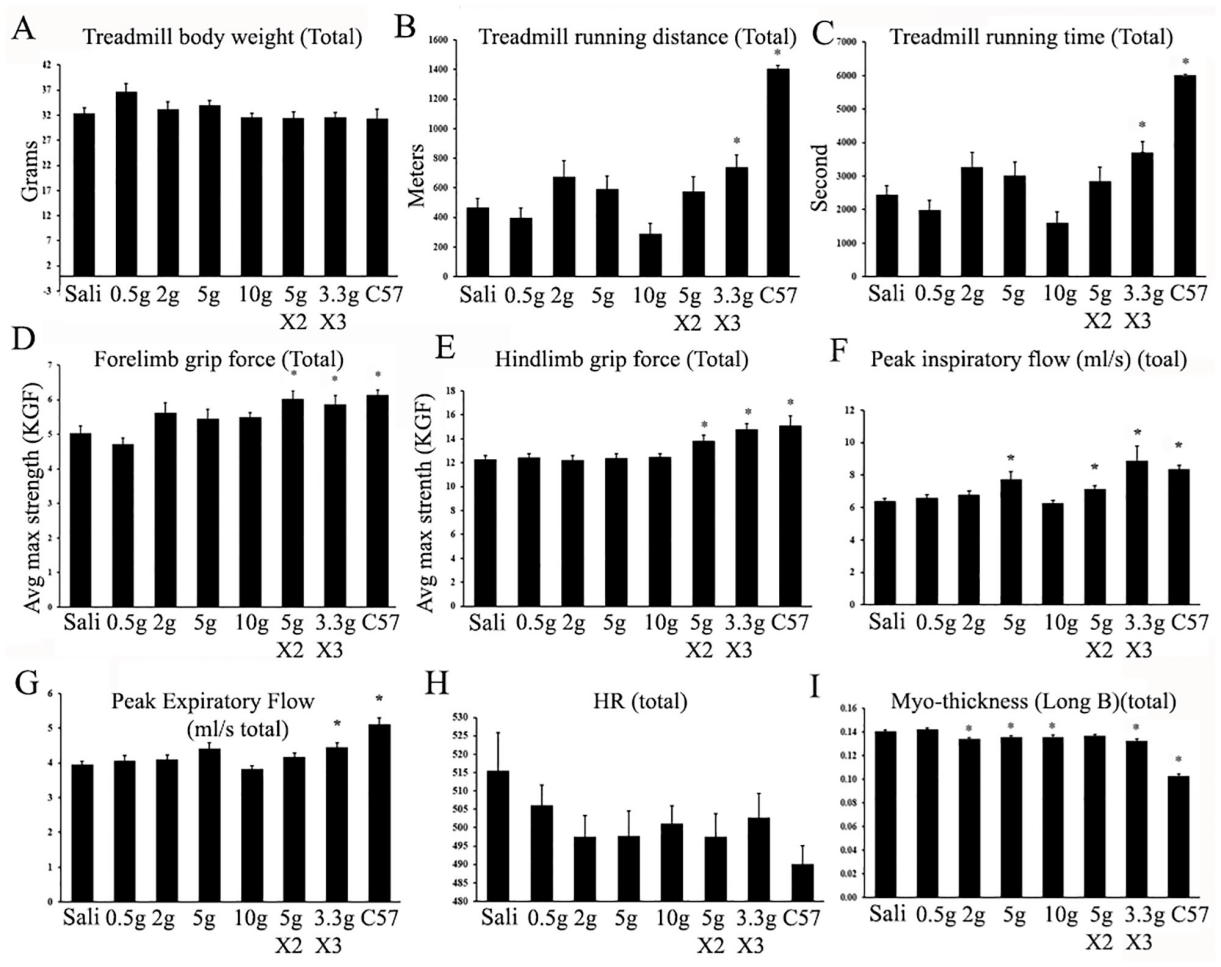
Effect of 6 month ribitol treatment on muscle functions. Skeletal muscle functions were measured by treadmill (A-C) and grip force (D-E). Respiratory and cardiac functions were measured by plethysmography (F-G) and echocardiography (H-I). Sali, saline-treated; 0.5g, 0.5g/kg; 5gX2, 5k/kg twice a day; 3gX3, 3g/Kg 3 times a day. * *p<0*.*05 n = 20* when compared with saline-treated *P448L* control.

Six-month treatment of ribitol did not affect the body weight significantly although 0.5g/kg group showed slightly higher mean body weight than all other groups. Treadmill exhaustion tests showed no significant difference between saline group and 0.5g/kg ribitol group. In contrast, except for the 10g/kg single delivery group, all other 4 groups with 2g/kg and higher doses of ribitol improved running distance and time and the 3.3g/kg 3 times delivery group showed statistical significance (Fig 4A–4C). The decrease in treadmill performance in the 10g/kg single delivery group was associated with strong GI side effect including chronic diarrhea (loose stool) which likely affects exercise performance, especially in endurance. Similarly, grip force strength was also significantly improved for the groups of 5g/kg twice a day and 3.3g/kg 3 times a day regime (Fig 4D and 4E). For respiratory function, significant improvement was also observed in Peak Inspiratory Flow (PIF) and Expiratory Flow (PEF) for 5g/kg or higher dose groups (Fig 4F and 4G; S4 Fig in S1 File). Importantly and consistent with decrease in fibrosis, improvement in cardiac function was indicated by reduction in heart rate and myocardium thickness with 4 groups of 2g/kg or higher dose treatment (Fig 4H and 4I; S5 Fig in S1 File).

### Side effects of ribitol treatment

Ribitol treatment was well tolerated with body weight remaining consistent with all groups of mice during the treatment period. H&E staining showed that kidney, liver, and spleen from all treatment groups were histologically normal (S6 Fig in S1 File). The only obvious side effect was the persistent GI bloating and diarrhea with severity being dose dependent. The most severe GI symptom was seen with 10g/kg single gavage whereas the same daily dose administrated across 2 and 3 times a day showed milder reaction. Symptoms of GI reaction almost disappeared 48 hours after the last treatment (S6 Fig in S1 File). Measurement of serum markers for liver and kidney showed no difference between treated and saline control mice (S7 Fig in S1 File). Interestingly, levels of LDL, ALT, total bilirubin and creatinine were reduced towards normal levels in the groups treated with ribitol.

### Detection of ribitol-phosphate and CDP-ribitol in serum and muscles of *P448L* mice after 6 months ribitol treatment

**Fig 5:**
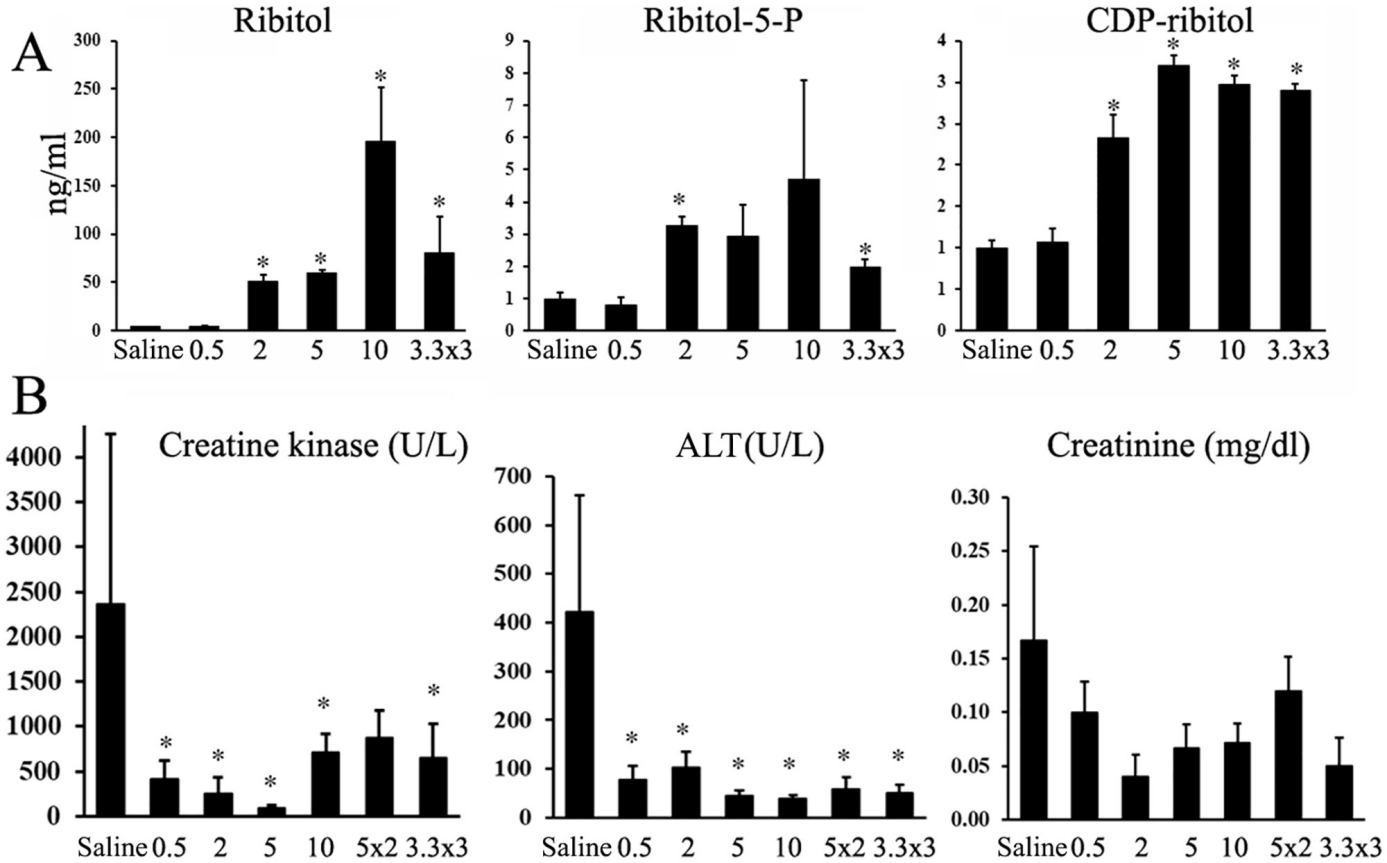
Detection of ribitol, ribitol-5-phosphate and CDP-ribitol and serum creatine kinase. (A) Levels of ribitol, ribitol-5-phosphate (P) and CDP-ribitol in quadriceps muscles 24 hours after last dose of ribitol treatment. Saline treatment as controls. Numbers on X axis are g/kg; x2, 2 times a day; x3, 3 times a day. (B) Ribitol treatment from 10 months of age decreases levels of serum creatine kinase and ALT. *, *p≤0*.*05 n = 3 (A) or n = 10 (B)*.

Our early study showed that levels of ribitol, R-5-P and CDP-ribitol in muscles of *P448L* mice increased after 5% ribitol treatment in drinking water [20]. To assess the dosing effect on these metabolites, we measured levels of the 3 metabolites in skeletal, cardiac muscle and serum 24 hours after the last gavage of ribitol. Levels of ribitol in serum was clearly dose dependent whereas both R-5-P and CDP-ribitol were only detected in the highest dose group (S8 Fig in S1 File). Similarly, levels of the three metabolites increased dose dependently in both cardiac and skeletal muscles, but levels of CDP-ribitol appeared to plateau at about 5g/kg daily dose in both muscles (Fig 5A and S8 Fig in S1 File).

### Ribitol treatment starting at 10 months of age dose-dependently improves muscle function

To assess the effect of ribitol on later stage LGMDR9, we treated *P448L* mutant mice starting from 10 months of age when significant fibrosis, especially in the diaphragm, and functional defects of skeletal muscles have progressed to an advanced stage. Importantly, our early follow-up of *P448L* mutant mice showed that almost all female mice died within 81 weeks of age [23]. We therefore extended the treatment and monitored the mice until they either manifested humane endpoint criteria (i.e., severe weight loss, deteriorating body condition, or inability to rise/ambulate) or reached a predetermined terminal endpoint of 2 years of age. To make relatively meaningful assessment of ribitol treatment on muscle function of the older mice, we performed skeletal, respiratory and cardiac function evaluation 6 months after initiation of the treatment when no mouse had expired in any experimental group.

Six-month ribitol treatment decreased the levels of creatine kinase and ALT in all dose groups with ribitol treatment (Fig 5B). The treatment did not change body weight significantly for most dose regimes except for the group with 0.5g/kg ribitol which has higher body weight than the saline control. No other side effect was recorded except that soft stool persisted and mild GI bloating was detected at the termination with daily doses of ribitol higher than 5g/kg.

**Fig 6:**
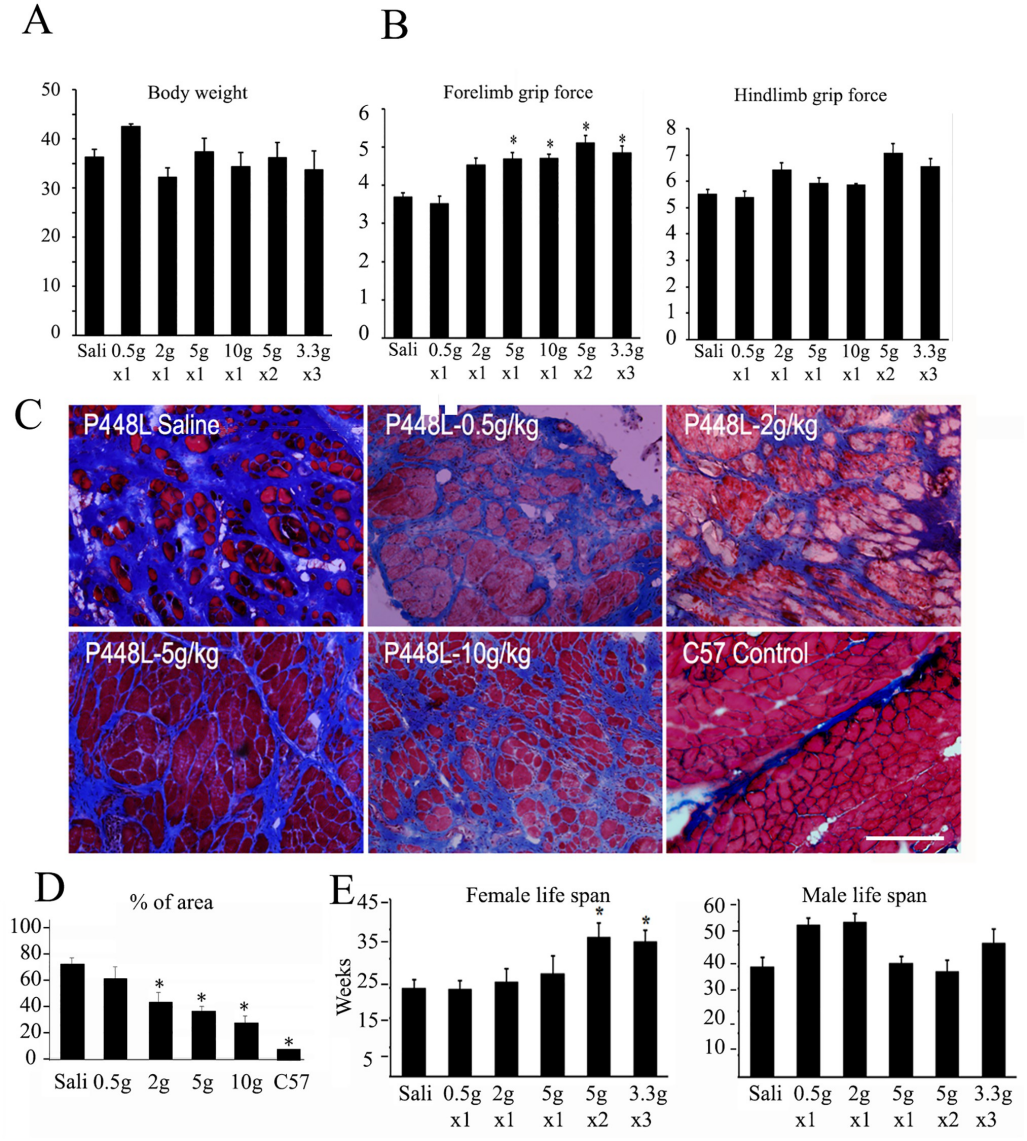
Effect of ribitol on body weight, muscle function, fibrosis and life span of *P448L* mutant mice treated with ribitol starting from 10 months of age. (A) Effect of ribitol treatment on body weight. (B) Ribitol effect on muscle function with grip force tests. (C) Masson Trichrome staining showed fibrotic area (represented by blue staining). (D) Measurement of Masson Trichrome staining of fibrotic areas. % of total area with blue staining. (E) Effect of ribitol treatment on life span of female and male mice. *, *P<0*.*5 n = 20* for A, B; *n* = 5 for D; *n = 10* for E. Sali, saline treatment as control. The number of weeks is calculated starting from the treatment at 10 months of age.

Muscle function measurement showed a significant improvement in forelimb grip force for the mice treated with ribitol of 5g/kg and above although hindlimb force did not change significantly (Fig 6A and 6B). Both running distance and time with treadmill exercise were not significantly changed between treatment groups and the control except for the group treated with 10g/kg ribitol (S9A Fig in S1 File), probably due to the very later stage of the disease, which was close to the end of their life expectancy. Echocardiography showed no significant changes except that left ventricular posterior wall end diastole (LVPWd) was consistently shorter in the ribitol treated groups than the control, suggesting less hypertrophy with ribitol treatment (S9B Fig in S1 File). Similarly, no significant changes were detected in respiratory function measured by plethysmography (S10 Fig in S1 File).

Histochemical analysis of the muscles from the mice terminated 2 weeks after the last functional examination with IIH6 antibody detected a dose-dependent increase in the levels of matriglycan (S11 Fig in S1 File). This enhancement was easily identified in all the muscles examined from the mice with 5g/kg or higher ribitol groups. Consistently, H&E staining show reduced fibrosis in TA, cardiac muscle, and especially diaphragm in the groups receiving 5g/kg or higher doses of ribitol (S12 Fig in S1 File). This was further confirmed by Masson Trichrome staining as illustrated in the Fig 6C and 6D.

### Life span of ribitol-treated *P448L* mice

Consistent with our previous report, all control *P448L* mutant female mice died earlier than 81 weeks with a mean survival age of 64 weeks, significantly shorter compared to their male counterpart (Fig 6E). Ribitol treatment of 0.5g/kg did not improve life span. However, treatment with daily doses of 2g/kg and above increased life span of the female mice, reaching statistical significance with the two highest dose groups of 10g/kg daily ribitol (mean survival age of 76 weeks) when compared to the control female group (Fig 6E). Life span in the male *P448L* mice treated with ribitol was similar to the saline control, most of them living longer than 80 weeks.

## Discussion

Experimental therapy with the aim to restore matriglycan on α-DG for *FKRP*-related diseases by AAV-mediated gene therapy has been reported with high efficacy in preventing disease from progression in mouse models [7, 24, 25]. However, clinical trials of the therapy for the diseases with such a wide range of phenotypes are challenging and remain to be conducted. We reported earlier that, ribitol, a natural pentose alcohol delivered by drinking water *ad libitum* can restore therapeutic levels of matriglycan and ameliorate dystroglycanopathy caused by FKRP *P448L* mutation associated with severe CMD phenotype in clinic [20, 26]. In this preclinical evaluation, we determined the minimum effective dose of ribitol under a clinically applicable treatment regime with once and three times daily gavage. Wide-spread enhancement of matriglycan expression and some improvement in muscle functions with statistical significance were achieved with daily single dose delivery of 2g/kg body weight. Daily dose of 10g/kg showed better improvement in matriglycan expression and muscle function. However, dividing 10g/kg daily dose into 2 and 3 times for delivery appeared to have limited benefit, except that the GI side effect was clearly milder than single delivery. GI response to pentitol is likely species specific, and therefore may not be directly relevant to clinical application [27]. Nevertheless, the degree of improvement in both matriglycan expression and muscle function is similar between 5g/kg and 10g/kg groups, suggesting a possible saturation of ribitol treatment. This is also supported by the levels of CDP-ribitol which are similar in muscle samples from groups treated with 5g/kg and 10g/kg. This, however, will unlikely limit dosing in human as the highest dose is practically difficult to achieve in clinics when the dose conversion rate from mouse to human is applied. The following factors could be important when considering dosing of ribitol in future clinical trials. First, *P448L* mutant FKRP retains possibly minimum residual function indicated by the lack of matriglycan in all muscle fibers except for a few newly regenerated revertant fibers, and by its association with CMD in clinics. The overwhelming majority of *FKRP*-related muscular dystrophy are LGMDR9 with mutations such as the common L276I mutation retaining considerably higher function as indicated by residual amounts of matriglycan and milder disease phenotypes when compared to *P448L* mutant [5, 21]. Second, our understanding that ribitol effect on matriglycan relies on residual function of FKRP in general, relative higher amount of residual matriglycan in diseased muscles might predict a higher efficiency with ribitol for inducing matriglycan. Therefore, effective dose range for treating LGMDR9 could be lower than that converted from the results of the current mouse tests. However, it should also be pointed out that different missense mutations may affect FKRP function with divergent mechanisms, from alteration in protein transportation to enzymatic activity. Thus, the degree of efficacy with ribitol treatment for different mutations could only be established by trials in a patient population.

One apparent difference between ribitol treatment and AAV gene therapy is the variation in distribution of enhanced matriglycan. Determined by differential affinity of AAV serotypes to different muscle types and selective use of promoters in different tissues, AAV gene therapy is known to produce highly variable levels of transgene expression between muscles [7, 24, 28–31]. As an example, the most widely used AAV8 and AAV9 serotypes for muscular dystrophies have shown several folds higher levels of transgene expression in cardiac muscle than in skeletal muscles. This imbalance could have long term consequence for most muscular dystrophies associated with more severe disease phenotype of skeletal muscle than that of cardiac muscle. Therefore, high doses of AAV vector for achieving effective restoration of targeted protein in skeletal muscles could lead to over-expression of the targeted protein in cardiac muscle with undesirable consequences. Vice versa, AAV doses achieving effective restoration of targeted protein in cardiac muscle may be insufficient for effective rescue of skeletal muscles. Another important weakness for AAV-mediated gene therapy is the high variation in transgene expression even within a single muscle. This has been widely reported both from preclinical animal studies and clinical trials, especially when vector doses are not sufficiently high [7, 24, 31]. Different levels of transgene expression provide different degrees of functional enhancement and variable levels of protection from contraction-related damage to fibers within a single muscle, leading to higher liability to damage, and consequently continuous degeneration and loss of transgene. In contrast, ribitol treatment provides relatively lower levels of matriglycan restoration, but with highly homogenous distribution. This can be appreciated from immunohistochemistry showing more than 90% fibers being positive in all three representative muscles (heart, diaphragm and TA) of the ribitol treated mice with all dosages, and absence of fibers with strong membrane signal above overall signal intensity. This pattern of distribution is clearly different from AAV-mediated gene therapy which produced a mixed population of fibers with no, weak, and strong expression of matriglycan within a single muscle as we reported earlier [24]. A homogenous distribution of matriglycan expression would likely provide better protection and longer-term efficacy although short-term improvement in muscle function might not be as significant as high dose AAV gene therapy. Ribitol as a pentose metabolite is likely accessible to the cells in central nervous system (CNS) which is also affected by FKRP mutations although most the patients do not show overt manifestation. It will be interesting to determine if the same treatment could benefit the diseased CNS functionally and improve structural abnormality such as neuronal migration defect [32].

Mechanism(s) for shorter life span in untreated *P448L* mutant mice and significant increase in life span with ribitol treatment of female mice, but not male mice, are not understood. One possibility is that the shorter life-span of the female mice makes it easy for the study to demonstrate the benefit whereas such potential benefit could take much longer treatment and observation time to demonstrate in male mice. Ribitol as a natural metabolite and given relatively large amounts exogenously is expected to affect some steps of metabolic pathways as we demonstrated with the increase in R-5-P and CDP-ribitol in tissues and in serum. Understanding the mechanisms involved is important and our plan is to elucidate potential consequences of ribitol on metabolic pathways, especially the perceivable involvement in pentose phosphate pathway which is important for energy production, response to oxidative stress and building block production. It is worth noting that the levels of CDP-ribitol in the muscles appeared plateau after 5g/Kg treatment. CDP-ribitol is the final substrate for FKRP glycosyltransferase, and its level may well be critical for ribitol-induced enhancement in matriglycan. This might explain the limited further improvement by the dosage higher than 5g/Kg. Nevertheless, our current results provide sufficient preclinical efficacy and long-term safety data with suggested dosing and treatment regime for initial clinical trials.

In summary, this study demonstrates that ribitol is able to dose dependently increase matriglycan expression in clinically relevant FKRP mutant mouse model with a clinically applicable regime. Long-term ribitol treatment improves muscle pathology and function and increases life span without serious side effect. The results validate the mechanism that ribitol as a pre-substrate of FKRP glycosyltransferase is able to enhance the production of CDP-ribitol and compensate for the decreased function of mutant FKRP.

## Supporting information

S1 File (PDF)
