# Supplementary material for: Ribitol dose-dependently enhances matriglycan expression and improves muscle function with prolonged life span in limb girdle muscular dystrophy 2I mouse model
Source: PLoS One. 2022 Dec 1;17(12):e0278482. doi: 10.1371/journal.pone.0278482 (PMC9714851; doi:10.1371/journal.pone.0278482)

Supplementary information

Supplementary Figures

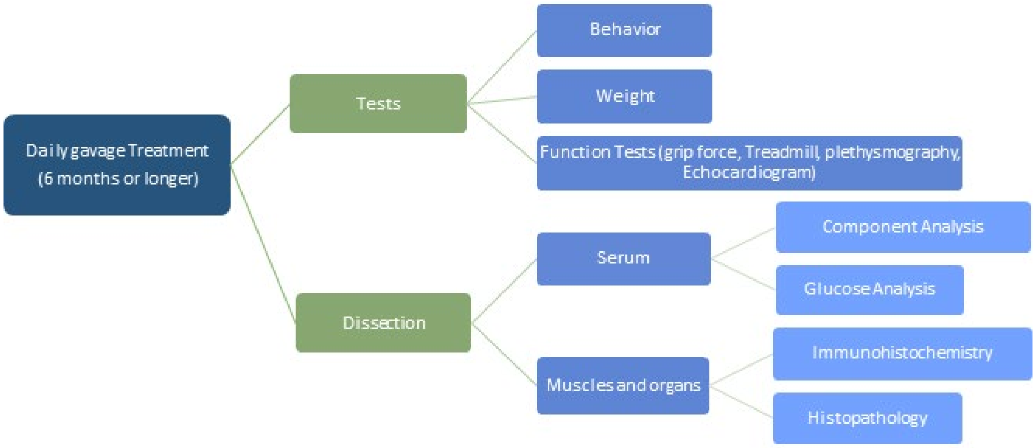

Supplementary Figure 1. Experimental procedure for testing efficacy of ribitol treatment by gavage in *P448L* mutant mice.

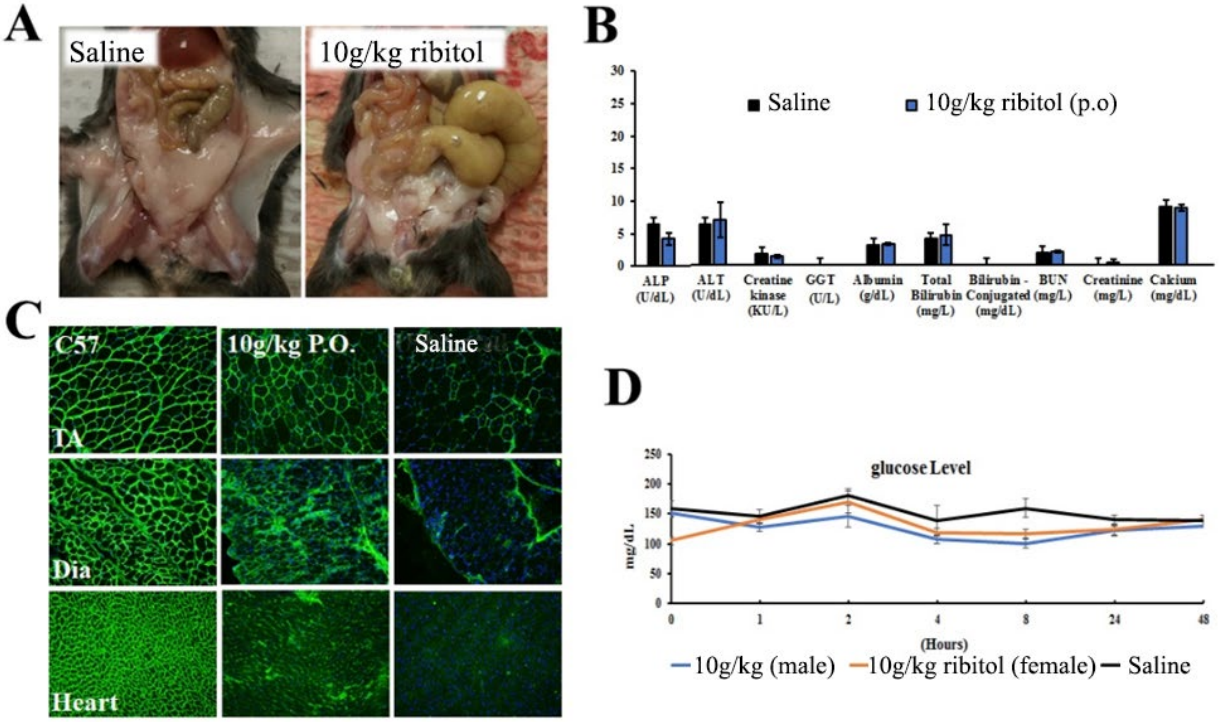

Supplementary Figure 2. **One-month treatments of *P448L* mutant mice with daily 10g/kg ribitol delivered by gavage (P.O.)**. (A) Gastrointestinal bloating is seen after 10g/kg ribitol treatment. (B) Serum test shows no significant difference between treated and controls. n=10. (C) Improved expression of matriglycan in the treated compared to saline controls in heart, diaphragm (Dia) and Tibialis Anterior (TA). Sections were stained with anti-matriglycan IIH6 and detected with AlexaFluor 488 goat-anti-mouse secondary antibody (green). Blue is nuclear counterstaining with DAPI. Scale bar, 150μM. (D) Blood glucose levels within 48 hours after single 10g/kg ribitol treatment. n=3. Saline treatment as controls.

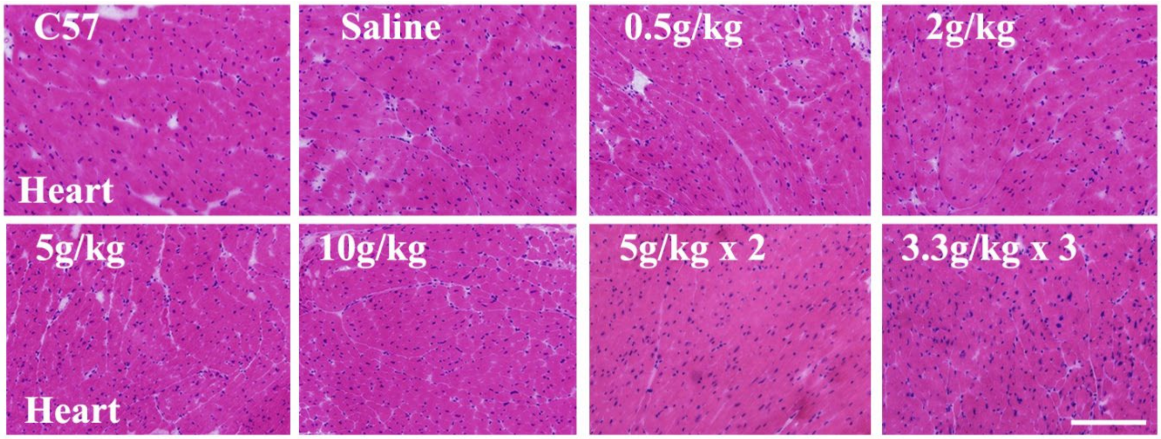

Supplementary Figure 3. **Cardiac muscle pathology of *P448L* mutant mice after ribitol treatment.** Saline treatment as control. Scale bar, 150μM.

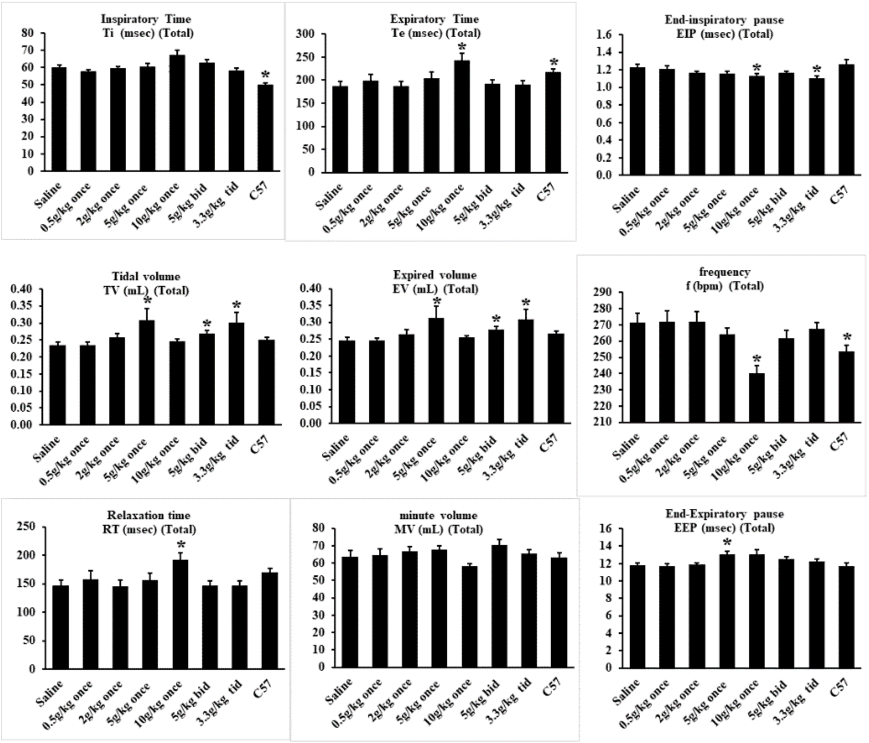

Supplementary Figure 4. **Measurement of respiratory function by plethysmography in *P448L* mutant mice after 6 months of ribitol treatment** (also refer to Figure 4). * $P<0.05$, n=20 when compared with saline treated *P448L* mutant mice.

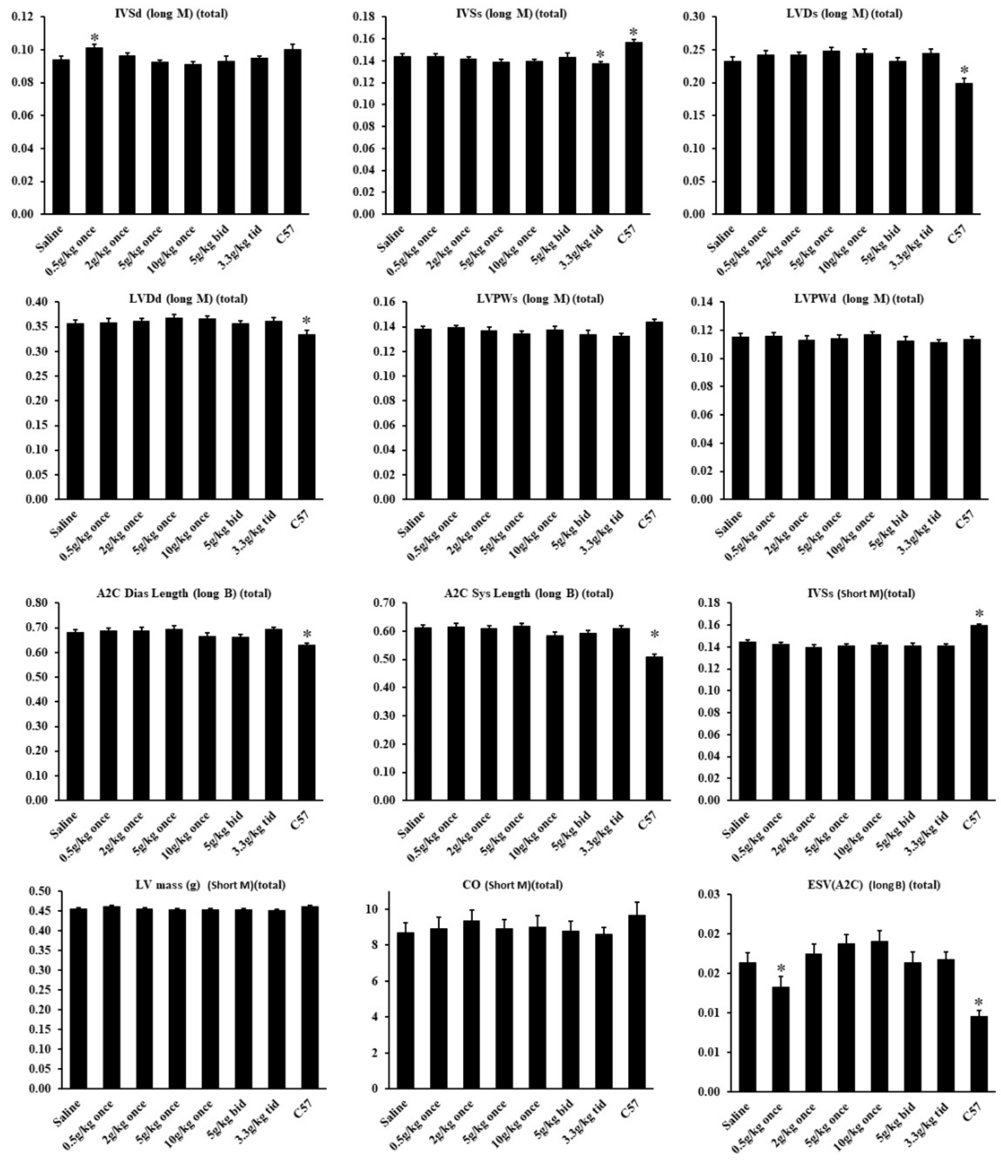

Supplementary Figure 5. Measurement of cardiac function in *P448L* mutant mice. Measurement was performed after 6 months of ribitol treatment by Echocardiogram using the Bioscan SonixTablet Ultrasound System (also refer to Figure 4). IVSd, Interventricular septum diastole; IVSs, Interventricular septum systole; LVDd, Left ventricular diameter diastole; LVDs, Left ventricular diameter systole; LVPWs, Left ventricular posterior wall systole; LVPWd, Left ventricular posterior wall diastole; A2C Dias, Apical two-chamber view diastole; A2C Sys, Apical

two-chamber view systole; IVSs, Interventricular septum systole; LV, Left ventricle; CO, Cardiac output; ESV, End systolic volume. * P<0.05, n=20

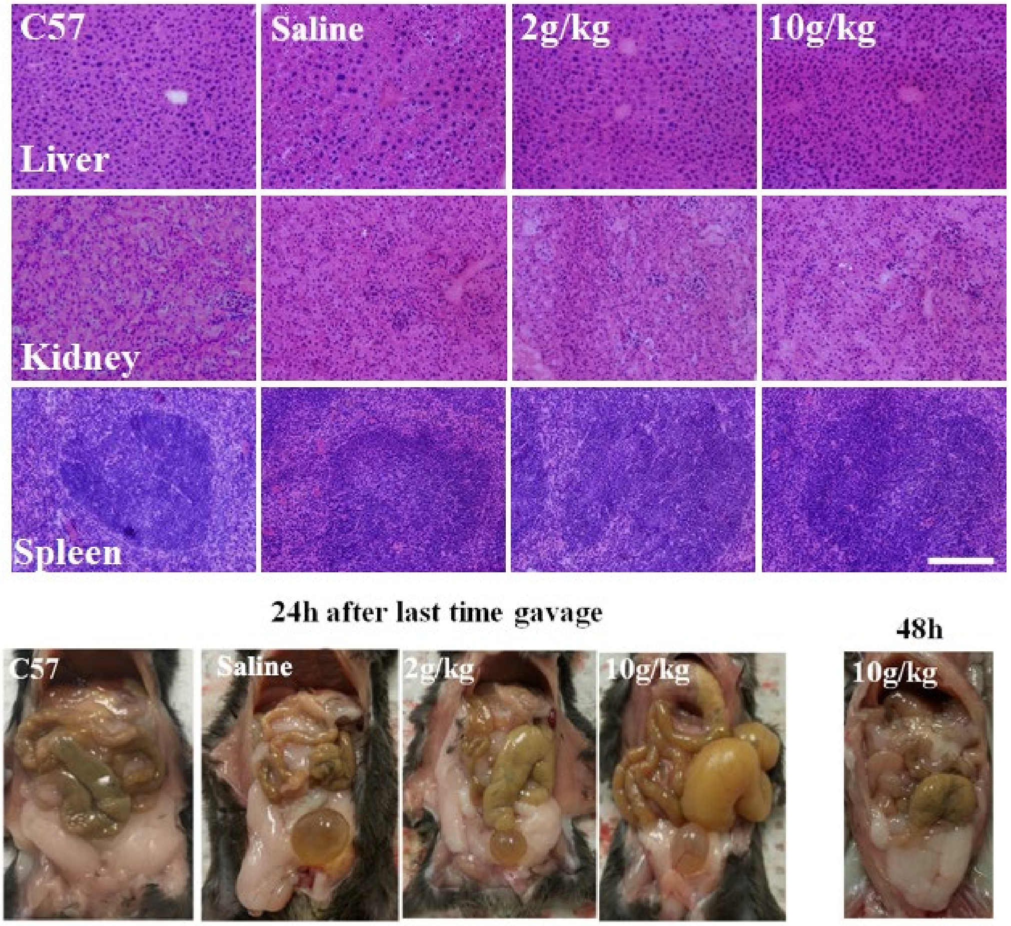

Supplementary Figure 6. Effect of 6-month ribitol treatment on *P448L* mutant mice. (A) H&E staining of liver, kidney and spleen from control C57, saline-treated control and 2g/kg and 10g/kg ribitol treated groups (Upper panel) Scale bar, 150μM. (B) Open view of abdomen of the mice from the different treatment and control groups. The most severe bloating is seen in large intestine of the mouse with 10g/kg daily single treatment. However, bloating almost disappears 48 hours after last treatment.

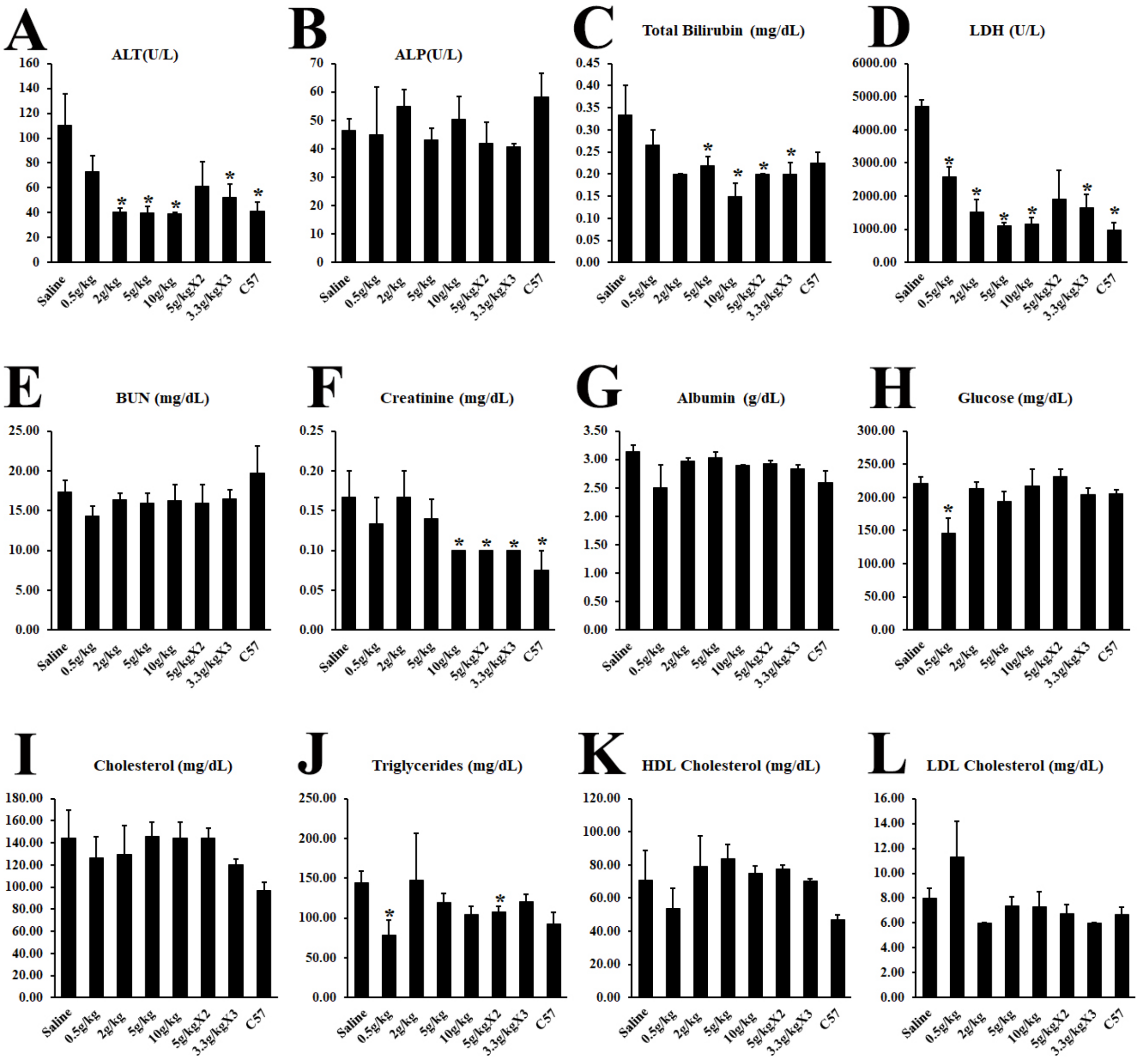

Supplementary Figure 7. **Measurement of serum markers after 6-month ribitol treatment in *P448L* mutant mice**. Saline treatment as control; *C57* controls. * P<0.05, n=10 when compared with saline treated *P448L* mutant mice.

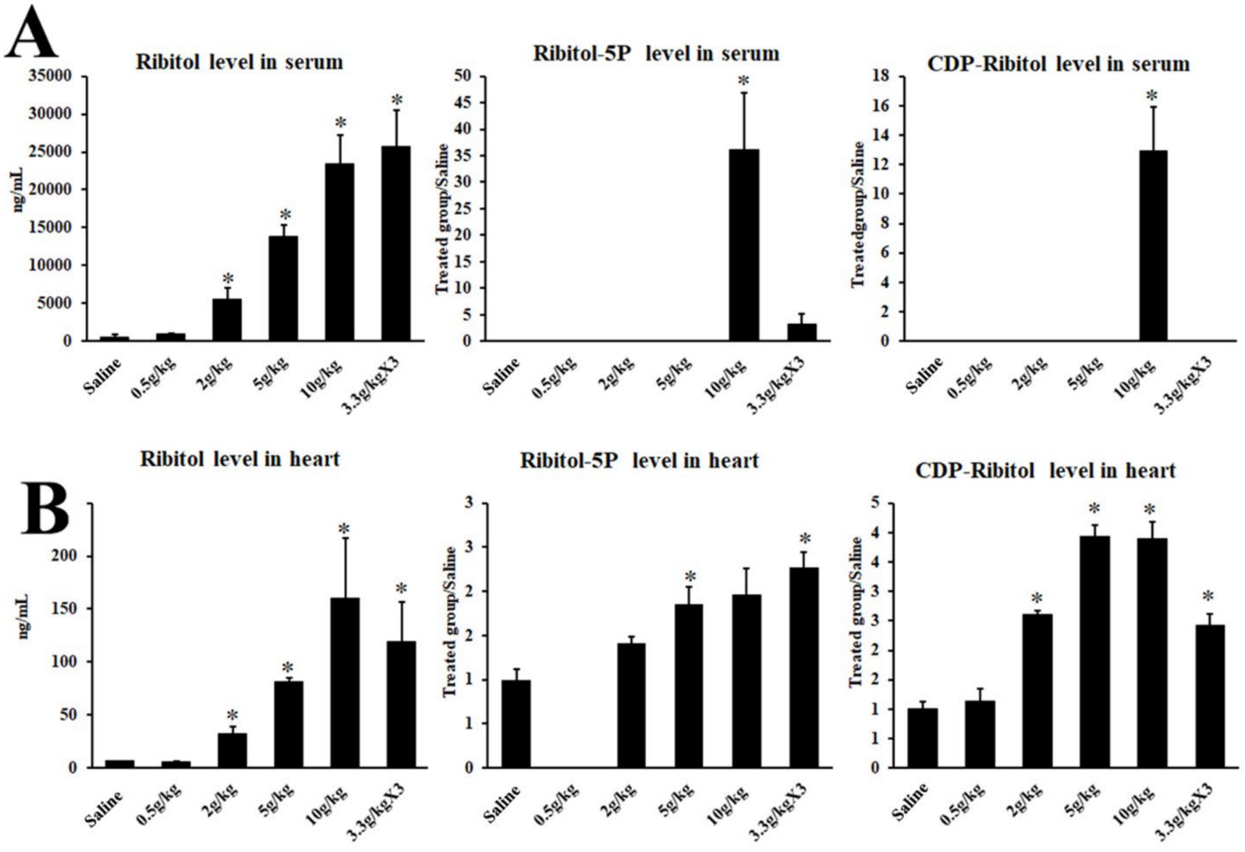

Supplementary Figure 8. **Detection of ribitol, ribitol-5-P and CDP-ribitol in serum and cardiac muscle tissues.** Tissues were prepared 24 hours after ribitol treatment (also refer to Figure 5 for measurement of the 3 metabolites in quadriceps muscles). * P<0.05, n=3 when compared with saline treated *P448L* mutant mice.

A

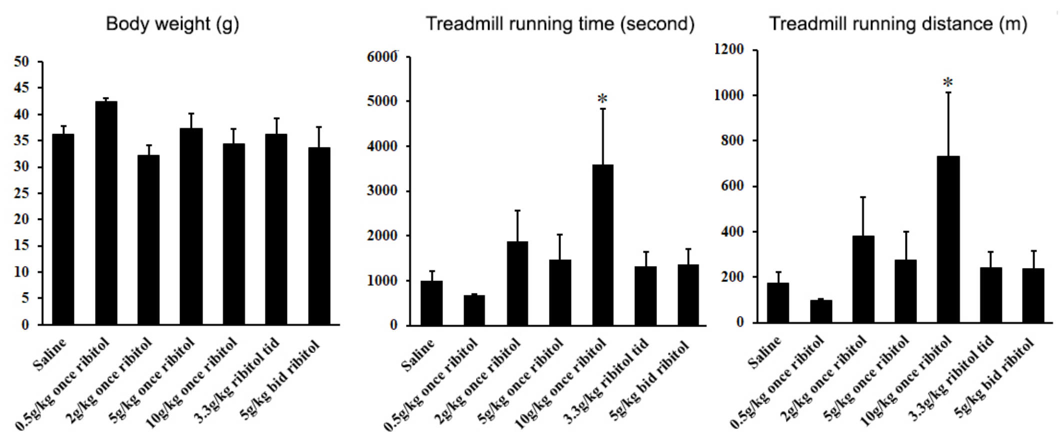

B

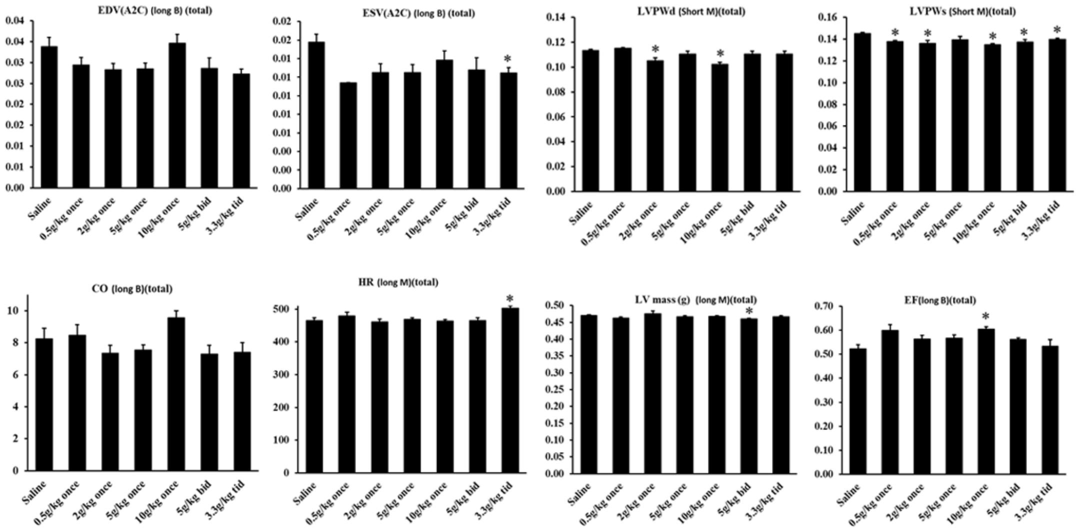

Supplementary Figure 9. Skeletal and cardiac muscle function of *P448L* mutant mice treated with ribitol starting from 10 months of age. (A) Body weight, and treadmill exercise time and distance measurement. (B) Measurement of cardiac function in *P448L* mutant mice with ribitol treatment starting from 10 months of age by Echocardiogram using the Bioscan SonixTablet Ultrasound System. EDV, End diastolic volume; ESV, End systolic volume; LVPWs, Left ventricular posterior wall systole; LVPWd, Left ventricular posterior wall diastole; CO, cardiac output; HR, Heart rate; LV,

Left ventricle; Ejection fraction (EF). * P<0.05, n=20 when compared with saline treated *P448L* mutant mice.

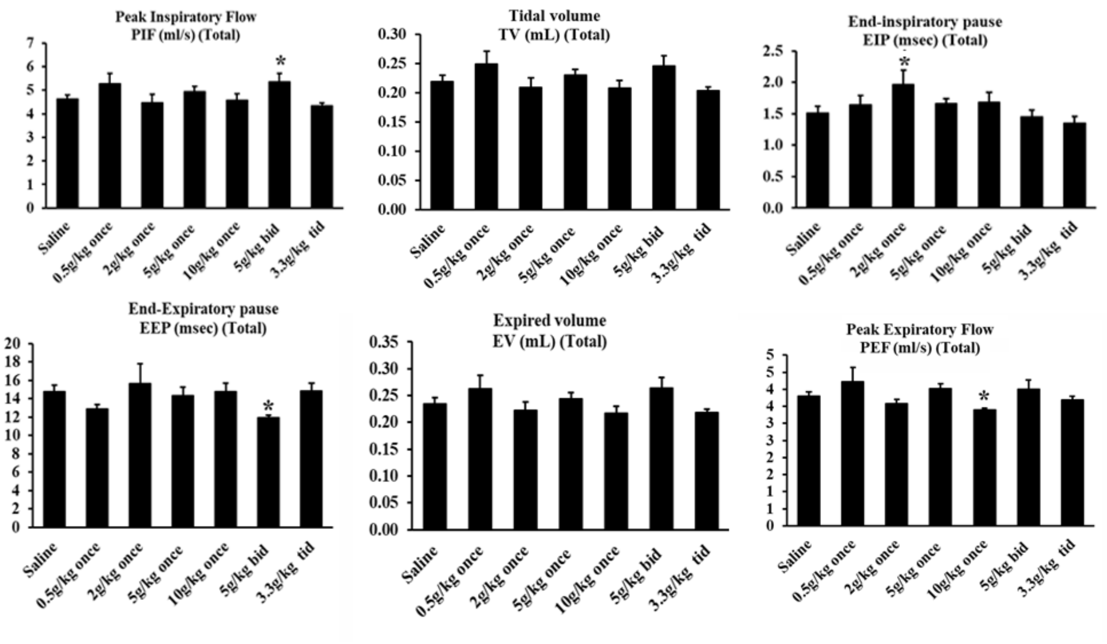

Supplementary Figure 10. **Measurement of respiratory function in *P448L* mutant mice with ribitol treatment starting from 10 months of age by plethysmography**. * P<0.05, n=20 when compared with saline treated *P448L* mutant mice.

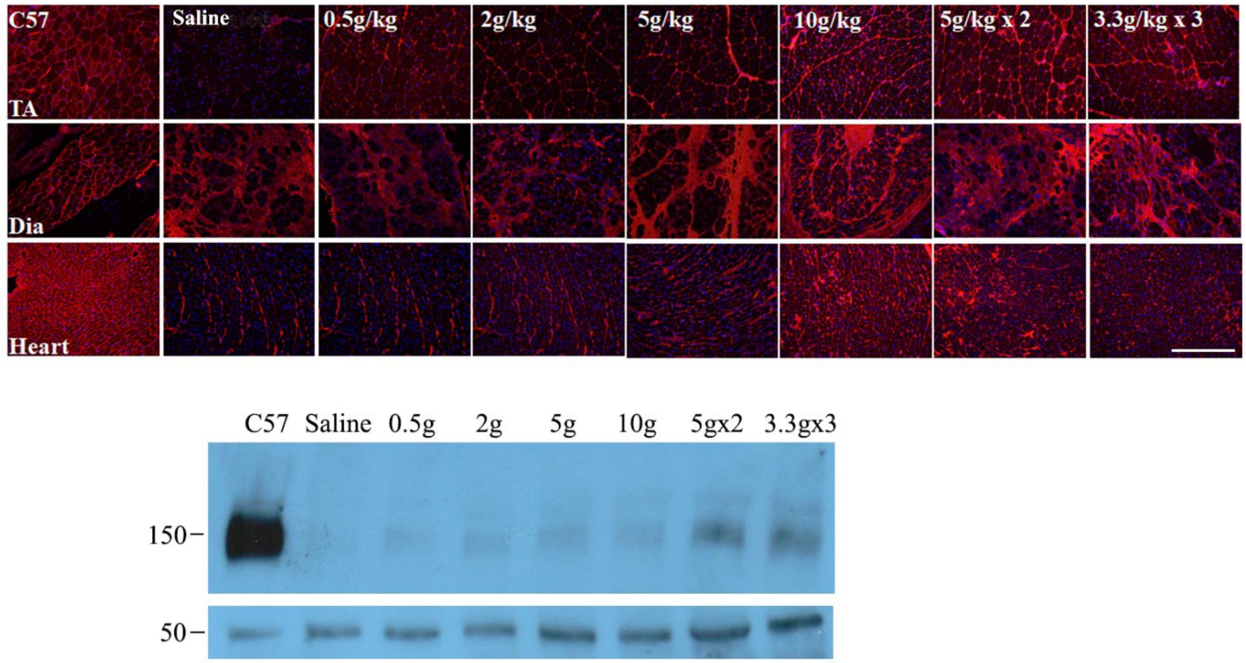

Supplementary Figure 11. Matriglycan detection in *P448L* mutant mice with 6-month ribitol treatment starting from 10 months of age for the expression of matriglycan. Upper panel, immunostaining of muscles shows a dose-dependent inclease in intensity of IIH6 staining in all the three muscles. Red staining represents expression of matriglycan. Blue, DAPI counter-staining for nuclei. Dia, diaphragm; Saline, Saline-treated control. Scale bar, 150μM. Lower panel, western blot detection of matriglycan with IIH6 antibody in Tibialis anterior muscles with the doses ranging from 0.5g/Kg (0.5g) single time a day to 3.3g/Kg 3 times (3.3gx3) a day. Actin is used as loading control.

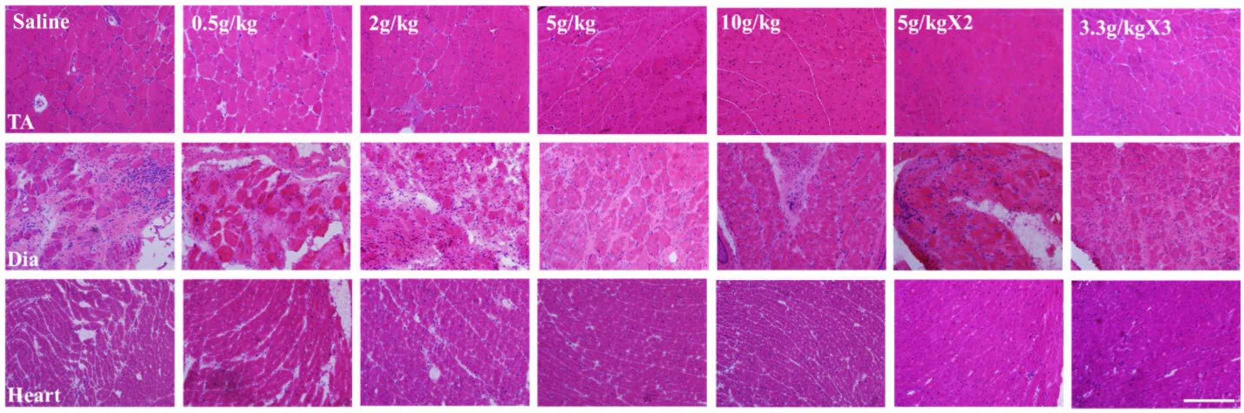

Supplementary Figure 12. H&E staining of the muscles from *P448L* mutant mice with 6-month ribitol treatment starting from 10 months of age. Ribitol dose-dependently improves muscle histology with decrease in fibrosis, especially in the diaphragm (Dia) when compared to the saline-treated control. Scale bar, 150μM.

Original images of the western blot for matriglycan detection in Figure 1

Figure 2 B, TA

C57

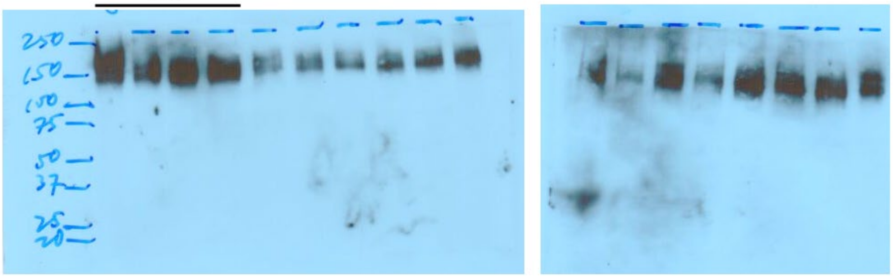

Figure 2 B, diaphragm

C57

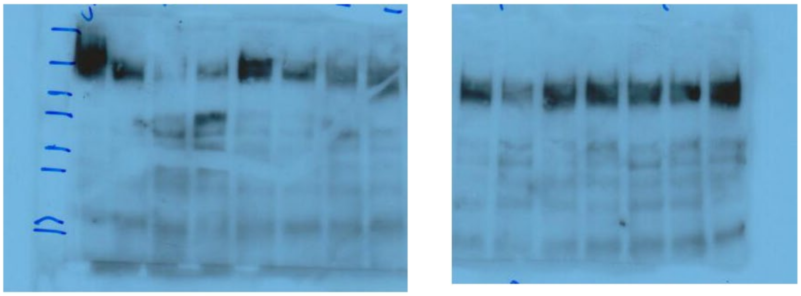

Figure 2 B, Heart

C57

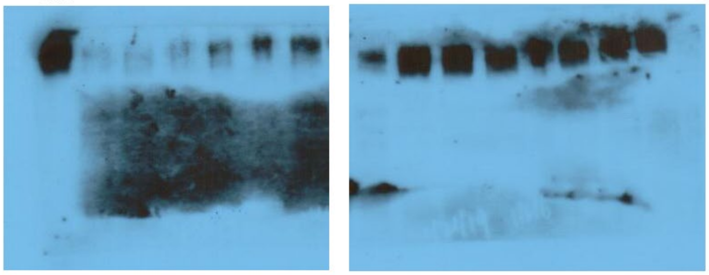

Supplement: S1 File — (PDF) [file pone.0278482.s001.pdf]
